# The Influence of Microsecond Pulsed Electric Field and Direct Current Electric Field on the Orientation Angle of Boron Nitride Nanosheets and the Thermal Conductivity of Epoxy Resin Composites

**DOI:** 10.3390/mi16040413

**Published:** 2025-03-30

**Authors:** Yan Mi, Yiqin Peng, Wentao Liu, Lei Deng, Benxiang Shu

**Affiliations:** State Key Laboratory of Power Transmission Equipment Technology, School of Electrical Engineering, Chongqing University, Chongqing 400044, China; pyq@stu.cqu.edu.cn (Y.P.); liuwentao@stu.cqu.edu.cn (W.L.); dl@stu.cqu.edu.cn (L.D.); 202411131288@stu.cqu.edu.cn (B.S.)

**Keywords:** EP composites, electric field orientation, thermal conductivity, local orientation, global arrangement

## Abstract

The electric field orientation method effectively promotes the orientation and arrangement of BN nanosheets, forming a thermal conduction network and enhancing the thermal conductivity of the composite material. In this study, microsecond pulsed electric field and direct current electric field were applied to induce the orientation and arrangement of BN nanosheets and improve the thermal conductivity of epoxy resin composites. Under a microsecond pulsed electric field of 50 Hz, 1.5 μs, and 8 kV/mm, the average orientation angle of BN nanosheets increased by 147.7%, and the thermal conductivity of the composite reached 0.352 W/(m·K), which is 1.84 times that of pure epoxy resin. In contrast, under a DC electric field of 70 V/mm, the average orientation angle of BN nanosheets increased by only 57.9%, while the thermal conductivity of the composite reached 0.364 W/(m·K), 1.91 times that of pure epoxy resin. The results indicate that the microsecond pulsed electric field primarily enhances the local orientation of the fillers to improve thermal conductivity, whereas the DC electric field mainly enhances the global arrangement of the fillers to achieve a similar effect. Additionally, thermogravimetric analysis and differential scanning calorimetry were conducted to evaluate the thermal properties of the composites. The results demonstrate that after BN nanosheets orientation and arrangement within the epoxy resin induced by both microsecond pulsed and DC electric fields, the composites exhibited a higher glass transition temperature and improved thermal stability. This study systematically explores the effects of microsecond pulsed and DC electric fields on filler orientation and arrangement, providing valuable insights for the fabrication of electric field-oriented composites.

## 1. Introduction

With the rapid development of the power electronics industry, power electronic devices are advancing toward high integration and miniaturization. During operation, these devices experience a significant increase in power consumption and heat accumulation. If heat is not effectively dissipated in a timely manner, thermal failure may occur, affecting the stable operation and service life of the components. Epoxy resin (EP) is widely used in power system insulation and electronic device encapsulation due to its excellent electrical insulation, corrosion resistance, and other advantageous properties [1,2]. However, the inherently low thermal conductivity of pure EP results in poor heat dissipation performance, which accelerates aging when exposed to high temperatures over extended periods. This degradation ultimately shortens the service life of devices and may even lead to premature insulation failure [3,4,5]. Therefore, enhancing the thermal conductivity of EP to improve heat dissipation in electronic devices is a critical issue that needs to be addressed.

Filling polymer materials with high thermal conductivity fillers is a common method for enhancing their thermal conductivity [6,7,8,9]. Pawelski-Hoell et al. [10] filled boron nitride (BN) with different particle sizes and boehmite into a glass fiber-reinforced epoxy novolac matrix via a solvent-free impregnation method. This method enhanced the thermal conductivity of the composite material. For example, the in-plane thermal conductivity of the laminate containing 20 vol% BN 12-μm reached 0.665 W/(m·K), and the through-plane thermal conductivity reached 1.043 W/(m·K). Given a fixed filler size, shape, and content, regulating the orientation and arrangement of high thermal conductivity fillers within the matrix can enable the formation of an efficient heat conduction network along the heat flow direction, even at low filler concentrations. This approach is a decisive factor in improving the thermal performance of composite materials. Luo et al. [11] prepared a calcined silica/cationic polymer/nanodiamond (SD*@ND) filler with high electron affinity through self-assembled in situ polymerization, electrostatic interaction, and calcination techniques, and incorporated it into silicone gel. This method effectively enhanced the thermal conductivity of the composite material, which reached 0.557 W/(m·K), showing a significant improvement compared to commercial silicone gels. Wang et al. [12] proposed a method to construct a controllable liquid crystalline cross-linking network based on the synthesis of biphenyl epoxy monomers and the adjustment of the structures of curing agents and curing temperatures. This method enabled the resulting liquid crystalline EP film to have a thermal conductivity as high as 0.53 W/(m·K). Zhao et al. [13] constructed a three-dimensional thermally conductive network of two-dimensional boron nitride nanosheets (BNNSs) and zero-dimensional boron nitride microspheres (BNMSs) through a solution blending and curing process. This method significantly improved the thermal conductivity of the epoxy resin composites. For example, the thermal conductivity of the BNNSs/BNMSs/epoxy resin composite with a filler content of 30 wt% reached 1.148 W/(m·K). Studies have shown that reorienting a small amount (volume fraction less than 20%) of thermally conductive fillers to form interconnected bridges within the polymer matrix is an effective strategy for enhancing thermal conductivity [14,15,16,17]. Common techniques for achieving this include the hot-pressing method [18], the ice-templating method [19], the magnetic field orientation method [20], and the electric field orientation method [21].

The electric field orientation method is one of the most effective and direct approaches for orienting fillers within a polymer matrix to form a thermal conduction network [22,23]. Kinoshita et al. [24] induced the orientation and arrangement of alumina platelets of three different sizes in an EP matrix using an electric field. Their study revealed that the thermal diffusivity of electric field-oriented composites was significantly higher than that of non-electric field-oriented composites, and the improvement increased with larger filler sizes. Kim et al. [25] used an electric field to induce the vertical orientation and arrangement of BN particles modified with nano-TiO_2_ in a polyurethane acrylate matrix. Their findings showed that at a filler content of 20 vol%, the thermal conductivity of the oriented composite reached 1.54 W/(m·K), which was 1.9 times that of the non-electric field-oriented composites. Bi et al. [26] filled an EP matrix with BN and applied a direct current (DC) electric field to orient the BN within the matrix, fabricating an electric field-oriented composite. The study found that as the electric field strength increased, the orientation and arrangement of BN improved, leading to an increase in the thermal conductivity of the electric field-oriented composite. When the electric field strength reached 13.33 kV/m, the thermal conductivity of the oriented composite increased to 0.93 W/(m·K), which was 1.6 times higher than that of the non-electric field-oriented composites. Mi et al. [27] used a microsecond pulsed electric field to induce the orientation and arrangement of BNNSs and fabricated an EP composite. Their study demonstrated that the thermal conductivity of the composite increased with higher electric field strength, frequency, and filler content. At a filler loading of 10 wt%, the application of a 12 kV/mm, 100 Hz pulsed electric field for BNNS orientation resulted in a composite with a thermal conductivity of 0.588 W/(m·K), more than 200% higher than that of pure EP. Liang et al. [28] utilized a pulsed square-wave electric field to induce the orientation and arrangement of hexagonal boron nitride (h-BN) within an EP matrix and systematically investigated the effects of electric field strength, frequency, and duration on the composite’s thermal conductivity. Their results indicated that under a 10 wt% filler loading, applying a 200 V/mm, 0.001 Hz pulsed square-wave electric field for 1 h resulted in a composite with a thermal conductivity of 0.453 W/(m·K), which was 3.24 times that of pure EP and 1.94 times that of the non-electric field-oriented composites.

In this study, BNNSs were used as a thermally conductive filler within an EP matrix, and both microsecond pulsed electric field and DC electric field were applied to induce the orientation and arrangement of BNNSs, leading to the fabrication of low-filler, high-thermal conductivity composites. The effects of microsecond pulse field strength and DC field strength on the orientation angle of BNNSs and the thermal conductivity of the composites were investigated. Furthermore, different electric field treatments were explored to control the orientation and arrangement of BNNSs. The thermal stability of composites oriented under different electric fields was comprehensively evaluated, providing a guiding exploration for applying the electric field orientation method in other composite material systems.

## 2. Materials and Methods

The principle of the electric field orientation method for preparing oriented high-thermal conductivity composites is as follows: During the curing process under an applied electric field, BNNSs undergo polarization and are subjected to the electric field force (*F_e_*), viscous resistance (*F_v_*), and Brownian motion force (*F_t_*) [29,30,31]. The *F_e_* is expressed as shown in Equations (1a) and (1b) [30]:

(1a) $$F_{e}=\pi \varepsilon_{0}\varepsilon_{s}a^{3}{\left(\beta E\right)}^{2}$$

(1b) $$\beta =\frac{\varepsilon_{p}-\varepsilon_{s}}{\varepsilon_{p}+2\varepsilon_{s}}$$

where *ε*_0_ is the permittivity of free space, *ε_s_* is the relative permittivity of the matrix, *ε_p_* is the relative permittivity of BNNSs, *a* is the in-plane radius of BNNSs (unit μm), *E* is the applied electric field strength, *β* is the polarization factor of BNNSs.

The *F_v_* acting on BNNSs during the curing process is primarily related to the viscosity of the prepolymer, and its expression is given in Equation (2):

(2) $$F_{v}=6\pi \eta_{s}a^{3}\gamma$$

where *η_s_* is the viscosity of the BNNS/epoxy resin prepolymer during the curing process, (unit: Pa·s); *a* is the in-plane radius of BNNSs (unit: µm); and *γ* is the shear rate during viscosity testing (unit: s^−1^).

Due to the relatively high temperature during the epoxy resin curing process, BNNSs undergo thermal motion (Brownian motion). The *F_t_* is expressed as shown in Equation (3):

(3) $$F_{t}=K_{b}T$$

where *K_b_* is the Boltzmann constant, and *T* is the temperature (unit °C).

**Figure 1:**
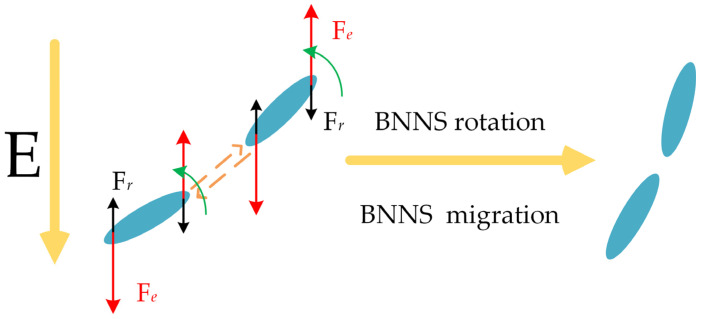
The process of BNNS rotation and approach under the electric field.

The electric field force promotes the rotational motion of BNNSs, whereas viscous resistance and Brownian motion force hinder their orientation. Therefore, BNNSs can only undergo rotational motion and align parallel to the pulsed electric field direction when the electric field force exceeds both the viscous resistance and Brownian motion force [29,30,31]. Furthermore, under the influence of Coulomb attraction, BNNSs are drawn toward each other, forming head-to-tail connections that establish heat conduction pathways, thereby enhancing the thermal conductivity of the composite material. The movement process is illustrated in Figure 1. In the figure, *F_r_* represents the hindering effect of viscous resistance and thermal motion on the orientation alignment.

### 2.1. Materials

**Figure 2:**
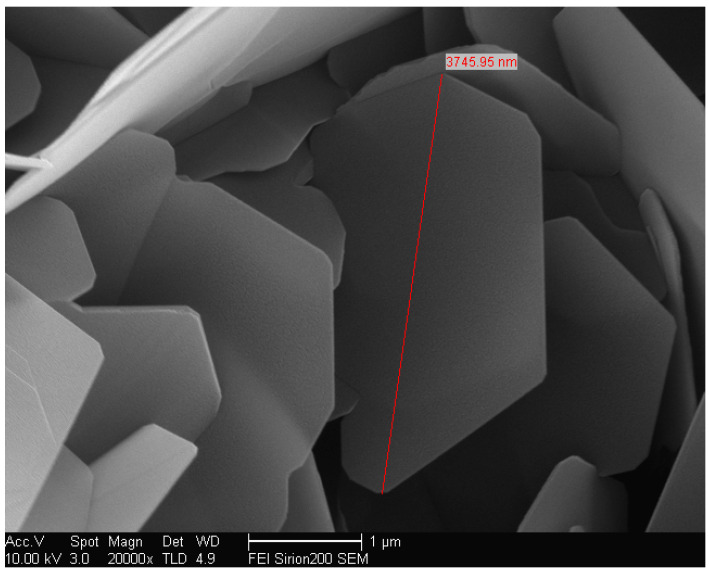
SEM micrograph of BNNS.

The EP used in this study was E-51, with methyl tetrahydrophthalic anhydride as the curing agent and DMP-30 as the accelerator, all purchased from Huakai Resin Co., Ltd., Jining, China. The filler used in this study was BNNSs with a thickness of approximately 50 nm and a diameter ranging from 0.5 to 5 μm, supplied by Beijing Deke Daojin Science and Technology Co., Ltd., Beijing China, without any surface modification. The SEM micrograph of the BNNSs is shown in Figure 2.

### 2.2. Sample Preparation and Orientation Method

**Figure 3:**
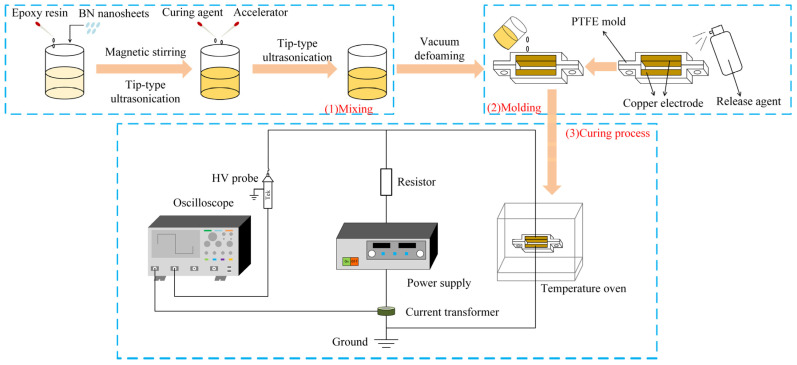
Schematic illustration of the preparation process for electric field-oriented composite.

Figure 3 illustrates the sample preparation process. First, EP and BNNSs were mixed together, with the BNNS loading in this study being 10 wt%. The mixture was then subjected to magnetic stirring for 1 h, followed by 30 min of ultrasonic treatment using a tip-type ultrasonic processor. Next, 8.5 g of curing agent and 0.2 g of accelerator were added to the mixture, which was further ultrasonically treated for another 30 min. The ultrasonically dispersed mixture was then vacuum-treated for 30 min to remove any trapped air bubbles. The obtained suspension was poured into a polytetrafluoroethylene (PTFE) mold. To ensure complete curing of the EP, the mold was heated at 90 °C for 2 h, followed by an additional 2 h at 110 °C [27]. Considering the effects of viscosity and time on the orientation of BNNSs, and to prevent molecular thermal motion from disrupting the thermal conductive network of BNNSs after orientation, and based on the preparation process in the reference, a pulsed or DC electric field was applied during the first 40 min of the curing process using two copper electrodes, with an electrode spacing of 1.4 mm [27].

**Figure 4:**
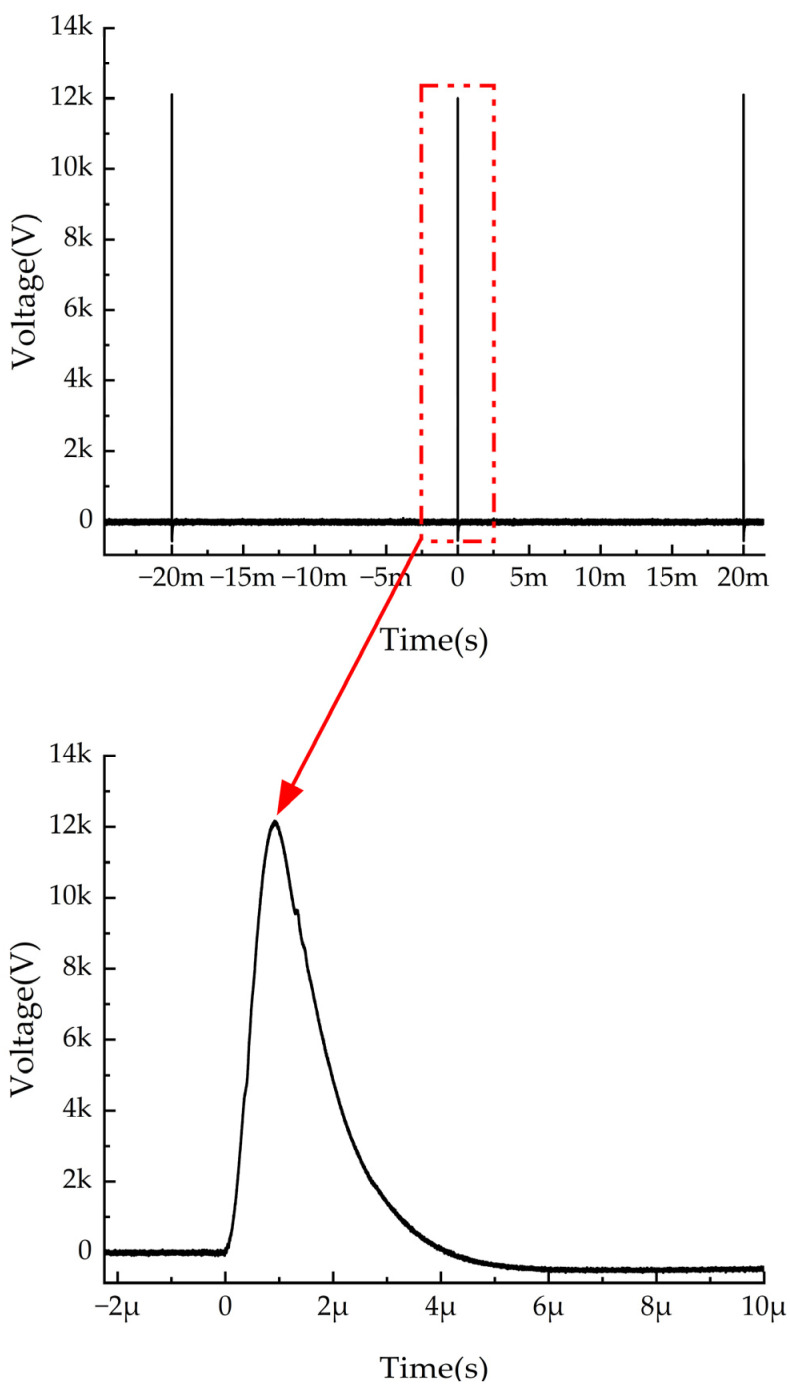
Waveform of the pulsed voltage.

The pulsed voltage used in this study was generated by a custom-built pulse generator in the laboratory. The waveform is shown in Figure 4, with parameters of 50 Hz, 1.5 μs, and 0–12 kV. For DC orientation, the applied voltage ranged from 0 to 120 V. However, preliminary experiments indicated that at higher DC orientation field strengths, the fabricated samples contained a significant number of voids, preventing further increases in the DC orientation field strength.

### 2.3. Characterization

The subjects of this study include four groups of materials: pure EP, EP composites without electric field orientation (EP/random BNNS composites), EP composites oriented by pulsed electric field (EP/oriented BNNS (with pulsed) composites), and EP composites oriented by direct current electric field (EP/oriented BNNS (with DC) composites).

The microstructure morphology of the sample’s surface and cross-section was observed using a scanning electron microscope (SEM) (Nova400, FEI Co., Hillsboro, Oregon, USA). Prior to observation, the samples were gold-sputtered. The orientation degree of BNNSs in the EP was characterized by X-ray diffraction (XRD) peak analysis using a PANalytical X’Pert Powder (Spectris Pte. Ltd., Almelo, Holland). The thermal conductivity of the composite material in the through-thickness direction was measured using a laser flash analyzer (LAF467HT, Netzsch Ltd., Bavaria, Germany) based on the laser flash method. The thermal stability of the composite was evaluated using a thermogravimetric analyzer (TGA) (TGA2, Mettler Toledo, Zurich, Switzerland). The glass transition temperature of the sample was tested using a low-temperature differential scanning calorimeter (DSC) (DSC3+, Mettler Toledo, Zurich, Switzerland).

## 3. Results and Discussion

### 3.1. Cross-Sectional SEM Images

**Figure 5:**
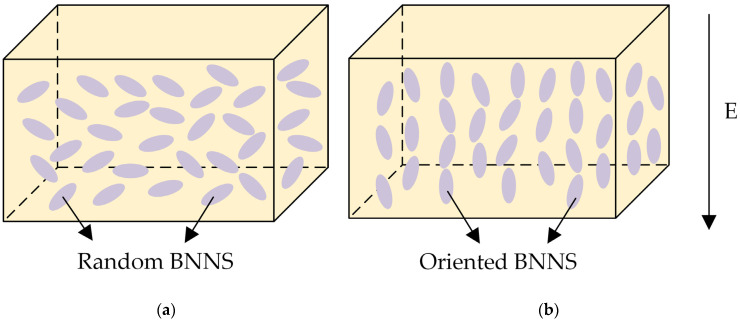
Schematic illustraion of BNNS distribution in the EP matrix: (**a**) without an orientation electric field; (**b**) with an orientation electric field.

Figure 5 illustrates the ideal distribution of BNNSs in the EP matrix for both EP/random BNNS composites and electric field-oriented composites. In an ideal scenario, BNNSs in EP/random BNNS composites are distributed in multiple directions, whereas in electric field-oriented composites, BNNSs should be oriented parallel to the direction of the applied electric field.

**Figure 6:**
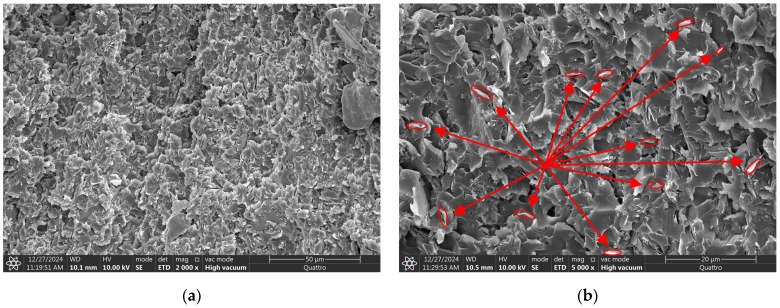

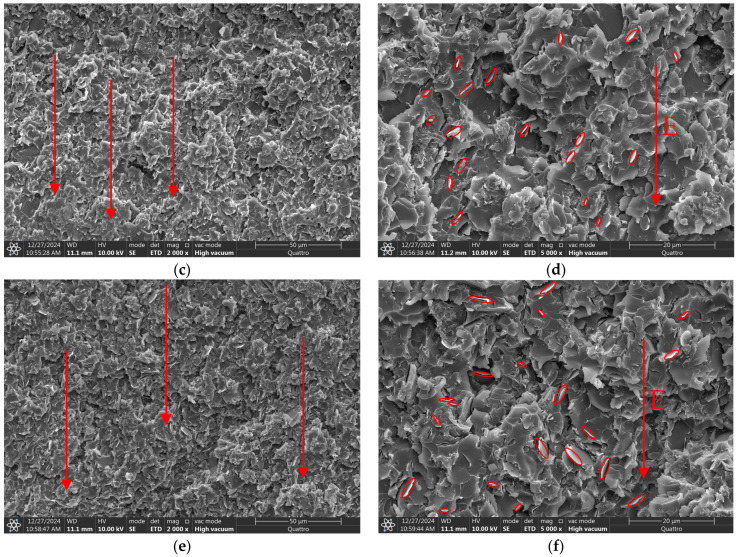
Cross-sectional SEM images: (**a**,**b**) BNNS in the EP/random BNNS composite; (**c**,**d**) BNNS in the EP/oriented BNNS (with pulsed) composite; (**e**,**f**) BNNS in the EP/oriented BNNS (with DC) composite. The arrows in the left figure represent the directional distribution of BNNS; these red circles represent BNNS and its angular distribution.

Figure 6 presents SEM images of the cross-sections of EP composites. In Figure 6a,b, which represent the EP/random BNNS composite, BNNSs are distributed in multiple directions. In contrast, Figure 6c–f show the EP/oriented BNNS (with pulsed) and EP/oriented BNNS (with DC) composites, respectively, where BNNSs exhibit a higher degree of orientation along the electric field direction. These results align with the theoretical distribution, and it can be observed that the EP/oriented BNNS (with pulsed) composite demonstrates a higher degree of BNNS orientation compared to the EP/oriented BNNS (with DC) composite.

### 3.2. XRD Patterns of Nanocomposites

Compared to SEM, which can only reflect the orientation results in local regions of BNNSs, XRD offers a larger detection area. The (002) crystal plane of BNNSs corresponds to the in-plane direction of BNNSs, while the (100) crystal plane corresponds to the thickness direction of BNNSs. Their diffraction peaks are observed at 2θ = 26.76° and 2θ = 41.60°, respectively. Therefore, the average orientation angle can be calculated more accurately based on the XRD test results.

**Figure 7:**
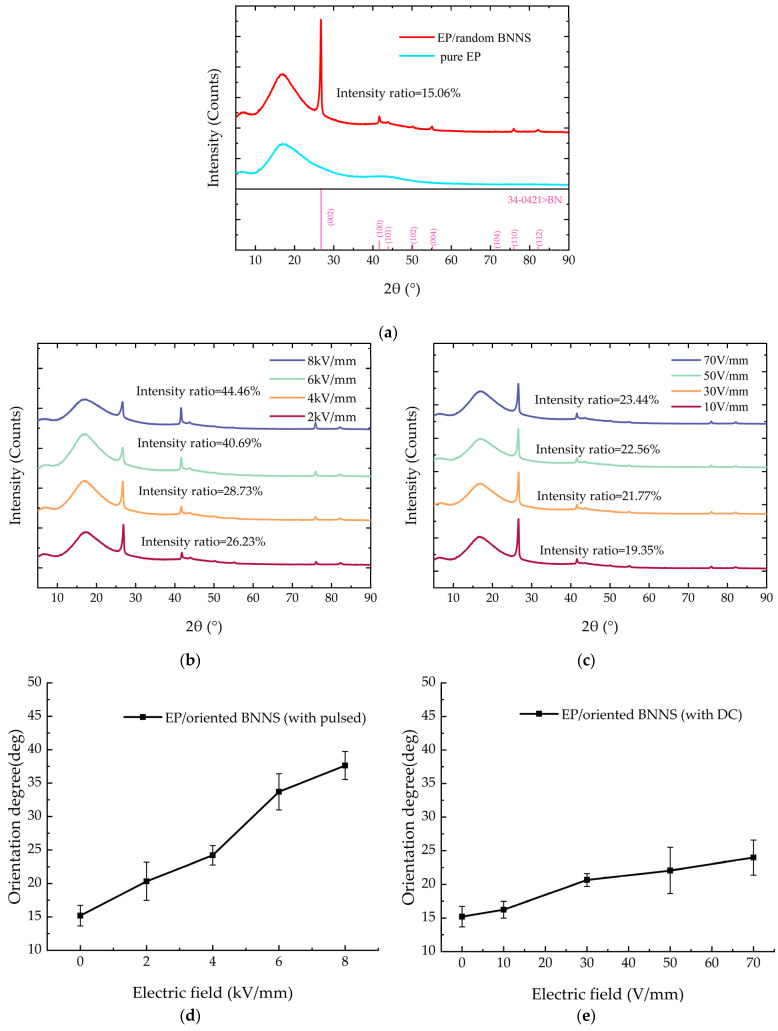
XRD patterns and average orientation angles of BNNSs under different electric fields: (**a**) XRD patterns of pure EP and EP/random BNNS composites; (**b**) XRD patterns of composites under different pulsed electric fields; (**c**) XRD patterns of composites under different DC electric fields; (**d**) Orientation angles of BNNS under different pulsed electric fields; (**e**) Orientation angles of BNNS under different DC electric fields.

The XRD patterns of pure EP, EP/random BNNS composite, and composites prepared under different pulse orientation field strengths and DC orientation field strengths are shown in Figure 7a–e.

From Figure 7a, it can be observed that compared to the diffraction peak pattern of pure EP, the EP/random BNNS composite exhibits multiple additional peaks within the range of 5°–90°. Notably, the peaks at 26.76° and 41.60° are more pronounced, and correspond to the intrinsic diffraction angles of BNNSs. This indicates that the newly introduced diffraction peaks in the composite originate from the incorporation of BNNSs.

The orientation degree can be estimated using the intensity ratio, as shown in the following Equation (4) [32,33]:

(4) $$\mathrm{Intensity}\mathrm{ratio}=\frac{I_{100}}{I_{002}+I_{100}}100\%,$$

Figure 7a–c show the intensity ratios of the diffraction peaks for different composites. The intensity ratio of the EP/random BNNS composite is only 15.06%. For the EP/oriented BNNS (with pulsed) composite, as the pulsed field strength increases, the intensity ratio of the diffraction peak gradually rises. When the applied pulsed field strength reaches 8 kV/mm, the intensity ratio increases to approximately 44.46%. For the EP/oriented BNNS (with DC) composite, the intensity ratio of the diffraction peak does not exhibit significant variation with increasing DC orientation field strength. When the applied DC electric field strength reaches 70 V/mm, the intensity ratio increases to approximately 23.44%.

The XRD pattern can reflect the BNNS diffraction peaks at different angles within the range of 5°–90°. To more comprehensively reflect the orientation degree of BNNSs, the average orientation angle $\bar{\varphi }$ can be calculated based on the XRD pattern using Equation (5) to fully evaluate the BNNS orientation degree [34,35]:

(5) $$\bar{\varphi }=\frac{\sum \left(I_{hkl}\times \phi_{hkl}\right)}{\sum I_{hkl}},$$

In the equation, $I_{hkl}$ represents the intensity of the diffraction peak, and $\phi_{hkl}$ is the directional angle, which is the angle between the (*hkl*) crystal plane and the (00L) basal plane. It can be calculated using Equation (6) [34,35]:

(6) $$\cos\phi_{hkl}=\frac{\frac{\sqrt{3}}{2c}l}{\sqrt{h^{2}+k^{2}+hk+\frac{3a^{2}}{4c^{2}}l^{2}}},$$

In the equation, *a* and *c* are the crystal parameters of boron nitride: *a* = 0.2173, *c* = 0.6657.

According to Equations (5) and (6), the average orientation angle of BNNS/EP composites prepared under different pulsed orientation field strengths and different DC orientation field strengths can be calculated, as shown in Figure 7d,e. From Figure 7, it can be observed that the average orientation angle increases with the increase in pulsed orientation field strength. Without an applied electric field, the average orientation angle of the EP/random BNNS composites is approximately 15.19°. When the pulsed orientation field strength reaches 8 kV/mm, the average orientation angle increases to around 37.63°, representing a 147.7% increase compared to the EP/random BNNS composites. For EP/oriented BNNS (with DC) composites, under the influence of a DC electric field, the average orientation angle of BNNSs shows minimal change, increasing only from approximately 15.19° to 23.99°. The main reason for the different average orientation angles of BNNSs under pulsed and DC electric fields is the applied field strength. Since the field strength of the DC electric field is much smaller than that of the pulsed electric field, it cannot provide an electric field force strong enough to overcome the viscous resistance and promote the rotation of BNNSs. This is the primary reason for the difference in the average orientation angles of BNNSs under the two types of electric field orientation.

Therefore, under the influence of the pulsed electric field and the DC electric field in this study, the pulsed electric field can effectively enhance the average orientation angle of BNNSs in the composite material, while the DC electric field has little effect on the average orientation angle of BNNSs.

### 3.3. Effect of Different Electric Field Orientations on the Thermal Conductivity of the Composite Material

This section will discuss the effect of different electric field orientations on the thermal conductivity of the composite material and explore the relationship between the average orientation angle and thermal conductivity, with the filler content fixed at 10 wt%.

**Figure 8:**
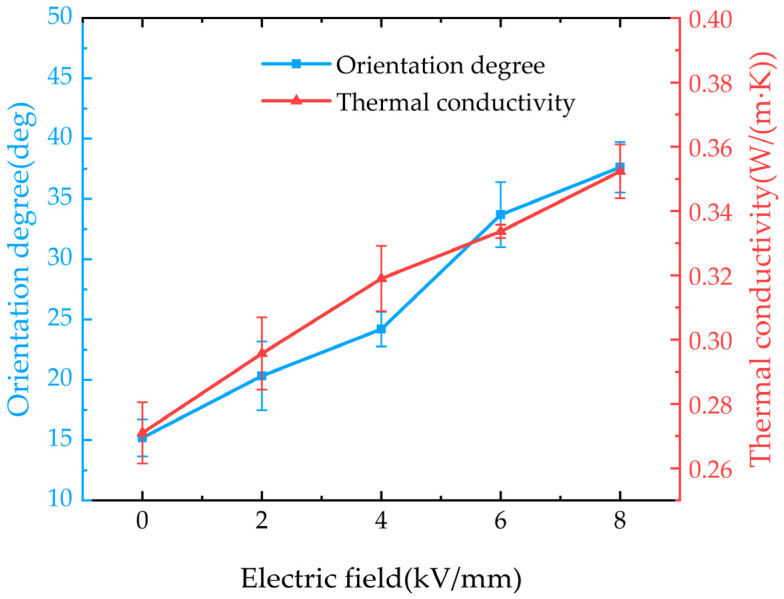
Thermal conductivity of composites and average orientation angles of BNNSs under different pulsed field strengths.

**Figure 9:**
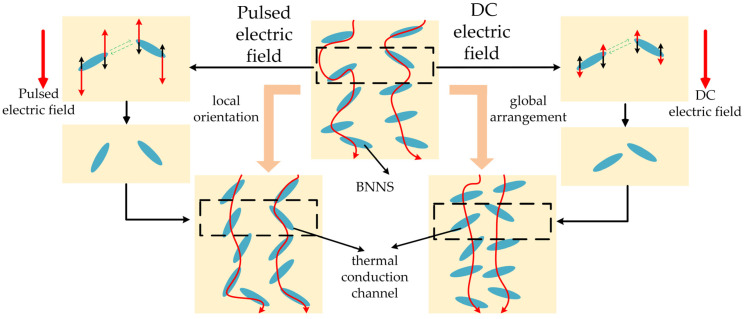
Schematic illustration of the ideal arrangement of BNNSs under pulsed and DC electric fields.

The thermal conductivity of the composite material under different pulse field strengths is shown in Figure 8. It can be observed that the thermal conductivity of the composite increases with the enhancement of the pulse orientation field strength. Under an 8 kV/mm pulse electric field, the thermal conductivity of the composite reaches 0.352 W/(m·K), which is 1.84 times that of pure EP (0.191 W/(m·K)). Combining the variation trend of the average orientation angle of BNNSs under the pulsed electric field, it can be observed that as the pulsed electric field increases, BNNSs reorient along the orientation direction, forming highly thermally conductive pathways in that direction, as shown in Figure 9. Therefore, the improvement in the thermal conductivity of the pulsed electric field-oriented composite exhibits a positive correlation with the average orientation angle of BNNSs.

Therefore, for pulse-oriented composite materials, the improvement in thermal conductivity is mainly due to the local orientation of BNNSs under the pulse electric field, which constructs an efficient heat conduction pathway in the orientation direction, as shown in Figure 9.

**Figure 10:**
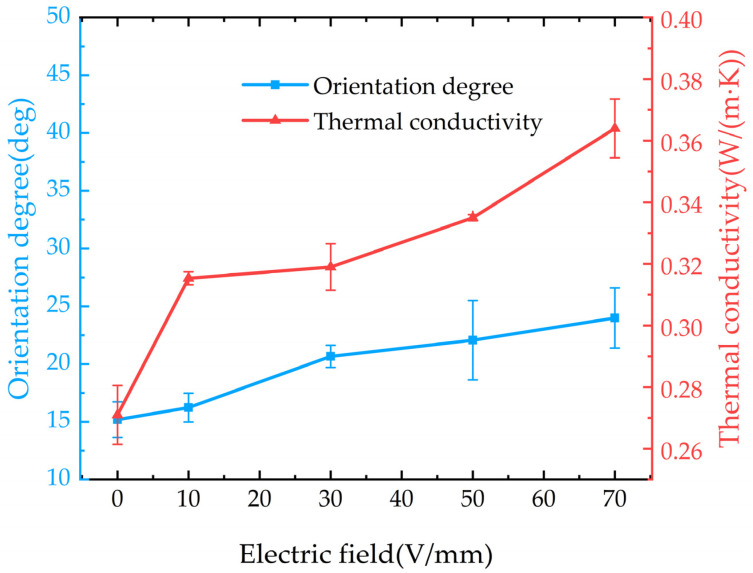
Thermal conductivity and average orientation angle of BNNSs in composites under different DC field strengths.

The thermal conductivity of the composites under different DC orientation field strengths is shown in Figure 10. It can be observed that the thermal conductivity of the composite increases with the enhancement of the DC orientation field strength. Under a DC electric field of 70 V/mm, the thermal conductivity of the composite reaches 0.364 W/(m·K), which is 1.91 times that of pure EP. By analyzing the relationship between the average orientation angle of BNNSs and the DC electric field, we find that there is little correlation between them. In the DC orientation field strength range of 30–70 V/mm, the average orientation angle of BNNSs does not increase significantly. However, the thermal conductivity of the composite still improves as the DC orientation field strength increases. Therefore, we can infer that under DC electric field orientation, the improvement in the thermal conductivity of the composite is not primarily due to an increase in the average orientation angle of BNNSs but rather due to the enhancement of the global arrangement of BNNSs, which brings them closer to each other, as shown in Figure 9.

Based on the analysis of the forces acting on the orientation of BNNSs and the results, it is evident that the pulsed electric field, due to its higher field strength, is more effective in enabling BNNSs to overcome viscous resistance and achieve local orientation, thereby forming efficient thermal conduction pathways. In contrast, the DC electric field provides a continuous field that induces a dipole coupling force, promoting the attraction between BNNSs, shortening the distance between high thermal conductivity BNNSs, and better facilitating the global arrangement of BNNSs, thus forming efficient thermal conduction pathways. Therefore, pulsed and DC electric fields construct thermal conduction pathways through different mechanisms of filler orientation and arrangement. The pulsed electric field primarily enhances the thermal conductivity of the composite by increasing the average orientation angle of BNNSs, while the DC electric field improves thermal conductivity by enhancing the global arrangement of BNNSs.

### 3.4. Thermogravimetric Analysis of Composites

To verify whether the pulsed and DC electric fields degrade the thermogravimetric performance of EP composites, this study compares only pure EP, EP/random BNNSs, composites prepared under a 50 Hz, 1.5 μs, 8 kV/mm pulsed electric field (EP/oriented with pulse (8 kV/mm)), and composites prepared under a 70 V/mm DC electric field (EP/oriented with DC (70 V/mm)). The BNNS filler content in the composites is fixed at 10 wt%.

**Figure 11:**
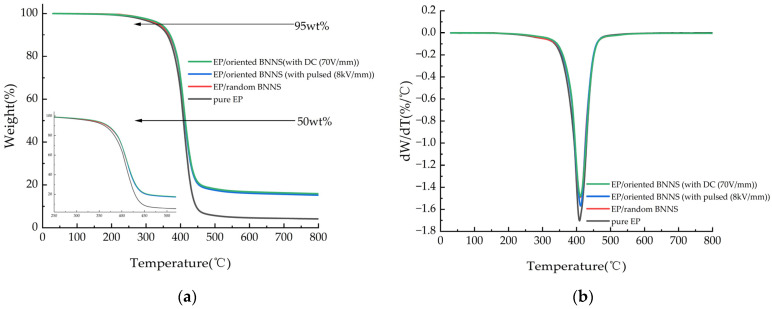
(**a**) TGA curves and (**b**) DTG curves of EP and its composites.

The thermogravimetric curves of pure EP and its composites under a nitrogen atmosphere, heated from room temperature to 800 °C at a rate of 10 °C/min, are shown in Figure 11a. The differential thermogravimetric (DTG) curves in Figure 11b represent the first derivative of the thermogravimetric curves. The temperatures corresponding to 5% mass loss (T_5%_), 50% mass loss (T_50%_), the maximum thermal decomposition temperature corresponding to the peak of the DTG curve (T_max_), and the residual mass percentage at 800 °C are listed in Table 1. These are common thermal stability parameters used to evaluate the thermal aging characteristics of composites.

From Figure 11, it can be observed that the thermal degradation behavior of the composite materials follows a similar trend to that of pure EP, indicating that the addition of BNNSs does not alter the thermal decomposition mechanism of the epoxy matrix. As shown in Table 1, the thermal decomposition temperatures of both EP/random BNNS and electric field-oriented composites are slightly higher than those of pure EP. This can be attributed to the inherently high heat capacity of BNNSs and their large, rigid sheet-like structure, which acts as an effective heat-resistant barrier. Additionally, the presence of BNNSs restricts the mobility of EP molecular chains, reducing flexibility and thereby delaying the thermal degradation process, ultimately increasing the decomposition temperature of the composites. As BNNSs undergo local orientation and global arrangement under the influence of the electric field, they progressively form a rigid chain structure, which further hinders the movement of EP molecular chains and enhances the thermal stability of the material. So, the thermal stability of electric field-oriented composites is slightly superior to that of EP/random BNNSs.

### 3.5. Glass Transition Temperature of Composites

**Figure 12:**
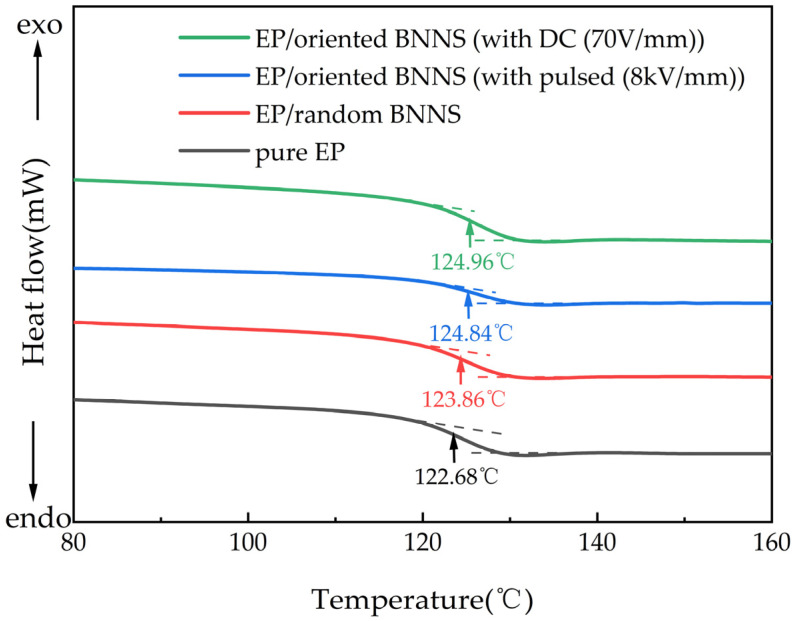
DSC curves of EP and its composites.

To verify whether the application of pulsed and DC electric fields affects the glass transition temperature of EP composites, differential scanning calorimetry (DSC) was performed on pure EP, EP/random BNNSs, EP/oriented BNNSs (with pulsed (8 kV/mm)), and EP/oriented BNNSs (with DC (70 V/mm)). The BNNS content in the composites was fixed at 10 wt%. Figure 12 presents the DSC curves of EP and its composites.

From Figure 12, it can be observed that the addition of BNNSs and the application of the orientation electric field slightly increase the glass transition temperature of the composite material. This is primarily due to the strong interfacial interactions between BNNSs and the EP matrix, which restrict molecular chain movement. Additionally, the steric hindrance effect of BNNSs forms physical barriers within the matrix, further limiting the rotation and mobility of molecular chains, thus requiring a higher temperature for the EP to transition from the glassy state to the rubbery state. Furthermore, during the curing process, the application of the orientation electric field facilitates the orientation and arrangement of BNNSs, leading to the formation of a skeleton-like rigid chain structure. This structure further enhances the restriction on molecular chain rotation and movement. Therefore, the incorporation of BNNSs and the orientation electric field can enhance the glass transition temperature of the composite to a certain extent, improving its stability under high-temperature conditions [36]. So, the glass transition temperature of electric field-oriented composites is slightly higher than that of EP/random BNNSs.

In summary, the incorporation of BNNSs and the application of an orienting electric field can enhance the thermal decomposition temperature, thermal stability, and glass transition temperature of the composite material without degrading its thermogravimetric performance.

## 4. Conclusions

In this study, BNNSs were oriented using both microsecond pulsed electric field and DC electric field to fabricate EP. SEM and XRD analyses confirmed that both types of electric fields induced the orientation and arrangement of BNNSs within the composite. Through quantitative analysis of the average orientation angle of BNNSs and the thermal conductivity of the composites, it was found that the pulsed electric field primarily enhances thermal conductivity by increasing the average orientation angle, while the DC electric field improves thermal conductivity by promoting the global arrangement of BNNSs. Other similar research results are summarized in Table 2. Compared to the research results of other scholars, although the thermal conductivity of the composites prepared in this study does not exceed that of others, this study enhances the thermal conductivity of the composites under a relatively low orientation field strength. Additionally, it investigates the construction of thermal conduction pathways under different electric fields, providing guidance for future research. Additionally, thermogravimetric analysis (TGA) and differential scanning calorimetry (DSC) showed that the oriented composites exhibit higher thermal stability and glass transition temperatures.

Future work could build upon these findings by exploring different matrix materials and filler characteristics, utilizing various electric fields to achieve optimized filler orientation and arrangement, and further enhancing the performance of composite materials in a targeted manner.

## Figures and Tables

**Table 1:** Thermal stability of EP and its composites.

Samples	T_5%_/°C	T_50%_/°C	T_max_/°C	Weight Loss Rate (800 °C)
Pure EP	329.167	408.500	408.833	94.860%
EP/random BNNS	336.500	413.167	410.167	84.398%
EP/oriented BNNS (with pulsed (8 kV/mm)	342.667	413.333	411.500	84.774%
EP/oriented BNNS (with DC (70 V/mm))	342.833	414.000	410.833	84.095%

**Table 2:** Other similar research results.

Samples	Type of Electric Field	Loading (wt%)	Electric Field Parameters	Thermal Conductivity W/(m·K)	References
the electric field-oriented EP/BN composite	DC	10	400 V/mm	0.544	[21]
	DC	25	13.3 kV/m	0.93	[26]
	PEF	10	11.76 kV/mm 100 Hz, 1 μs	0.588	[27]
	Pulsed Square-wave	10	200 V/mm, 0.001 Hz	0.453	[28]
	AC	10	120 V/mm, 50 Hz	0.310	[37]

## Data Availability

The original contributions presented in this study are included in the article. Further inquiries can be directed to the corresponding author.
